# Single-cell mapping reveals age-related alterations in periosteal progenitor cells and immune microenvironment

**DOI:** 10.1186/s13619-025-00263-9

**Published:** 2025-11-17

**Authors:** Lei Zhao, Chao Wu, Keran Chen, Zhaoning Xu, Yu You, Peiru Zhao, Di Zhu, Meiling Su, Jian Luo, Yiyun Wang

**Affiliations:** Shanghai YangZhi Rehabilitation Hospital (Shanghai Sunshine Rehabilitation Center), School of Medicine, Tongji University, Shanghai, China

**Keywords:** Periosteum, Aging, Bone homeostasis

## Abstract

Aging profoundly impacts bone homeostasis and regeneration, yet the cellular and molecular mechanisms underlying periosteal aging remain poorly understood. Using single-cell RNA sequencing, we profiled the periosteum of 3-, 9-, and 18-month-old mice, which revealed age-related shifts in progenitor, neutrophil, and macrophage subpopulations. Aging reduced mesenchymal cell populations and impaired osteogenic potential, may contribute to periosteal homeostasis. Periosteal progenitor subsets exhibited distinct aging trajectories: *Dpt*⁺ fibrous-layer cells undergoing early senescence, while *Postn*⁺ progenitors showed osteogenic decline. Aging also shifted immune profiles, increasing inflammatory *Cd38*^hi^ macrophages and dysfunctional *Nlrp3*^hi^ neutrophils, further disrupting bone homeostasis. Notably, aged progenitor cells upregulated CSF1 and CXCL signaling, driving macrophage and neutrophil infiltration, exacerbating bone loss. Our findings provide a comprehensive periosteal aging atlas, revealing aging-associated alterations in progenitor-immune crosstalk that may influence bone tissue dynamics, and offering insights into potential targets for age-related skeletal conditions.

**Supplementary Information:**

The online version contains supplementary material available at 10.1186/s13619-025-00263-9.

## Background

Aging disrupts skeletal metabolic homeostasis, heightens susceptibility to fragility fractures, and impairs fracture healing, collectively contributing to increased patient mortality (Kenzora et al. 1984). The periosteum, a dense membrane with an outer fibrous layer and an inner cambium layer, covers bone surfaces except at joints, tendon insertions, and sesamoid bones (Tang et al. 2023), playing a pivotal role in age-related skeletal pathologies. The vascularized cambium layer is enriched in bone progenitors, actively supporting bone growth and remodeling (Allen et al. 2004; Dwek 2010; Tang et al. 2023), whereas the fibrous layer, though less cellular, is densely packed with blood vessels and neural structures, providing mechanical support (Allen et al. 2004; Dwek 2010; Tang et al. 2023).

Periosteal skeletal stem cells (pSSCs) represent a distinct subpopulation within the periosteum that plays a vital role in bone homeostasis and fracture healing. Cambium-derived pSSCs contribute to homeostasis and early fracture repair, while a subset of fibrous layer-derived pSSCs, typically quiescent, are activated during late-stage healing, particularly in endochondral ossification (Liu et al. 2024). Although pSSCs are important, it is well recognized that multiple periosteal cell types—including various fibroblasts and immune cells—collaborate during the bone repair process. Despite the well-documented impact of aging on stem cells, the distinct age-related changes in periosteal stem/progenitor cells and their contributions to impaired bone homeostasis and regeneration remain poorly understood. Whether pSSCs from the cambium and fibrous layers exhibit differential aging responses is unknown. In addition to stem/progenitor cells, immune cells—particularly neutrophils and macrophages—are integral to bone homeostasis and regeneration, yet the influence of aged periosteal stem cells on innate immunity remains largely unexplored (Kovach et al. 2015; Medhat et al. 2019).

Here, we profiled the periosteum of young, middle-aged, and aged mice at the single-cell transcriptomic level, uncovering age-associated alterations in cellular composition, molecular function, and intercellular communication. Through these analyses, we aim to elucidate how these changes contribute to impaired bone homeostasis and regeneration with aging. Furthermore, we seek to identify key regulatory factors and signaling pathways underlying periosteal aging, offering new insights into potential strategies to mitigate periosteal deterioration and enhance bone repair.

## Results

### Dynamic single-cell aging atlas of the periosteum in mouse long bones

**Fig. 1:**
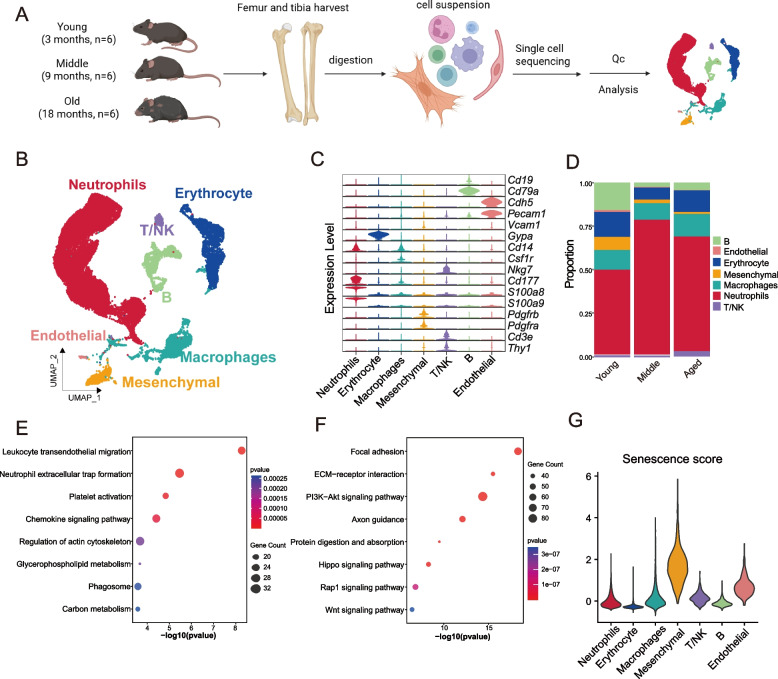
Preliminary analysis of single cell sequencing of periosteum in mice of different ages. **A** Schematic of the experimental workflow. Six mice, aged 3 months, 9 months, and 18 months respectively, were used for the dissection of the periosteum from long bones. **B** The UMAP plot displays various cell types identified through scRNA-seq, with colors indicating separate cell clusters. **C** Violin plots showing marker genes expression across various cell types. **D** Variations in cell type ratios across young, middle, and aged groups. **E–F** Analysis of KEGG pathway enrichment of DEGs (E) up-regulated and (F) down-regulated in aged periosteum compared to young periosteum. **G** The senescence scores of various cell subpopulations in the periosteum

To study age-related changes in the periosteum of long bones at the single-cell level, femurs and tibias of 3-, 9-, and 18-month-old mice (*n* = 6) were collected. The periosteal portion of the femurs and tibias was isolated for enzymatic digestion, and the resulting cell suspension was subjected to single-cell sequencing (Fig. 1A). After applying standard quality control criteria, including filtering based on gene count, UMI count, and mitochondrial content, a total of 32,931 cells were retained for downstream analysis, with 11,139 cells from the young group, 10,185 cells from the middle-aged group, and 11,607 cells from the aged group. Seven distinct cell types were identified based on known cell-type-specific markers including Neutrophils (*S100a8*^+^ and *S100a9*^+^), Macrophages (*Cd14*^+^ and *Csf1r*^+^), Mesenchymal cells (*Pdgfra*^+^), T/NK cells (*Nkg7*^+^), B cells (*Cd79a*^+^), Endothelial cells (*Cdh5*^+^ and *Pecam1*^+^) and Erythrocytes (*Gypa*^+^) (Fig. 1B-C). Given that senescent cells are characterized by specific markers, we examined two widely used senescence markers, *p21* (*Cdkn1a*) and *p16* (*Cdkn2a*), and found that *p21* was expressed in periosteal tissues. However, the expression level of *p21* did not exhibit a significant change with aging (Fig. S1A-B). Moreover, beta-galactosidase (β-gal) staining, a common indicator of senescence, was not detected in the periosteum of aged mice (Fig. S1C). These findings suggest that the aging process in the periosteum may require more precise identification methods to detect subtle senescence-associated changes.

To investigate age-related changes in the periosteum, the proportion of different cell subpopulations was first calculated. Previous studies have reported that during the aging process, stem cells in multiple tissues exhibit a decline in both their numbers and stemness (Brunet et al. 2023). Consistently, our study also observed a steady decrease in the proportion of mesenchymal cells with aging (Fig. 1D). Furthermore, we found that the proportions of neutrophils and macrophages were increased in the aged group compared to the young group, which was also confirmed by flow cytometry analysis (Fig. 1D, S1D-F). To further investigate age-related transcriptional changes in the periosteum, enrichment analysis was performed on DEGs between aged and young groups (Fig. S1G, Table S1). Pathways upregulated during aging included leukocyte transendothelial migration, neutrophil extracellular trap formation, platelet activation, and chemokine signaling (Fig. 1E), suggesting increased immune cell infiltration in the aged periosteum. Downregulated pathways during aging included focal adhesion, ECM-receptor interactions, PI3K-Akt signaling, and Wnt signaling (Fig. 1F). These downregulated pathways are closely associated with mesenchymal cell niche and osteogenic differentiation, highlighting the diminished bone regenerative potential of aged periosteum. In addition, we analyzed age-specific pathway alterations. Pathways such as oxidative phosphorylation, apoptosis, osteoclast differentiation, and the HIF-1 signaling pathway were specifically upregulated in the periosteum of 9-month-old mice compared to 3-month-olds (Fig. S1H). In contrast, pathways including Rap1 signaling, ferroptosis, T cell receptor signaling, and glucagon signaling were specifically upregulated in 18-month-old mice relative to 9-month-olds (Fig. S1I), indicating significant age-related changes in immune regulation and metabolic processes.

The senescence scores were calculated for periosteal cells using a published set of aging-related genes (Saul et al. 2022). We found that the senescence score of mesenchymal cells significantly increased with age, and among all cell types, mesenchymal cells exhibited the highest senescence score in the periosteum of 18-month-old mice. This suggests that mesenchymal cells undergo significant changes during the aging process (Fig. 1G, S1J). In addition, the senescence scores of neutrophils and macrophage populations gradually increase with age (Fig. S1J). The above results established a dynamic single-cell transcriptional profile of mouse periosteum and tentatively suggested that bone regeneration capacity may be diminished in aged periosteum.

### Alterations in cellular signaling networks during periosteal aging

**Fig. 2:**
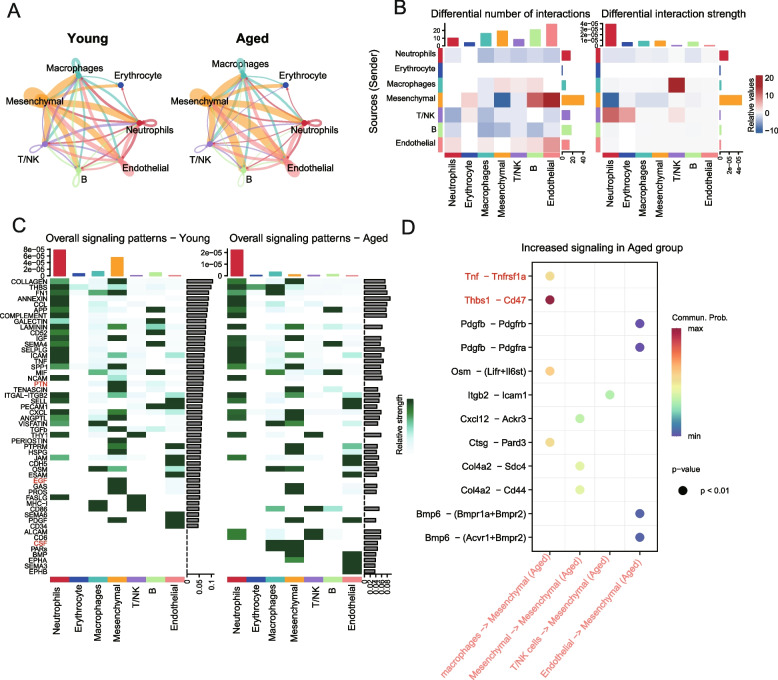
Changes in cellular communication in the periosteum during aging. **A** Quantitative comparison of the number of cell-to-cell communication events observed between the young and aged groups, highlighting differences in intercellular signaling networks associated with aging. **B** Analysis of age-related alterations in both the number and signaling strength (intensity) of cellular communications, comparing young and aged groups to reveal how aging affects intercellular interaction dynamics. **C** Changes in overall signaling patterns between aged and young groups. **D** The increased receptor-ligand interactions received by mesenchymal cells in the aged group compared to the young group

The periosteum consists of diverse cell types, whose complex interactions collectively regulate its function (Dwek 2010). To understand the complex regulatory network among these cells, CellChat (an R package designed for inference, analysis, and visualization of cell–cell communication from single-cell data) (Jin et al. 2021) package was used to analyze cellular communication. Among the different cell subpopulations in the periosteum, mesenchymal cells exhibit the most dominant interactions with other cell types. Regardless of age, mesenchymal cells maintain extensive cellular communication with other subpopulations in both the aged and young groups (Fig. 2A). Aged mesenchymal cells exhibited reduced autocrine signaling but enhanced crosstalk with endothelial cells. Notably, signaling from mesenchymal cells to neutrophils declined, whereas macrophage-to-T-cell communication was enhanced (Fig. 2B), revealing significant alterations in the cellular communication network during aging.

Overall signaling pattern analysis revealed that CSF signaling became highly enriched in macrophages and mesenchymal cells with aging (Fig. 2C), indicating that colony stimulating factor (CSF) signaling undergoes significant changes during periosteal aging and may play an important role. Conversely, pro-angiogenic and osteogenic signals, including epidermal growth factor (EGF) and pleiotrophin (PTN), were enriched in the young periosteum but diminished with age (Fig. 2C). This result is also consistent with the decline in osteogenic and angiogenic capacities during aging. In young periosteum, neutrophils and mesenchymal cells were dominant signal sources (Fig. S2A), whereas in aged periosteum, neutrophils remained the main recipients, while macrophages and mesenchymal cells exhibited reduced signal input (Fig. S2B).

The aging-induced alterations in signal reception by periosteal mesenchymal cells may detrimentally impact bone homeostasis and regeneration. Through receptor-ligand pair analysis of mesenchymal cells in young and aged states, it was found that aged mesenchymal cells exhibited increased THBS1-CD47 signaling from macrophages (Fig. 2D), a classical immune pathway previously linked to impaired muscle regeneration (Porpiglia et al. 2022), suggesting a similar role in osteogenesis. Additionally, TNF signaling from macrophages is significantly upregulated with aging, indicating an enhanced inflammatory response in aged periosteum (Fig. 2D).

In summary, our findings reveal age-related alterations in cellular communication among periosteal subpopulations. Specifically, the decline in vasculogenic and osteogenic differentiation signaling, alongside the upregulation of osteoclast signaling, constitutes the key communication changes associated with aging. These shifts likely underlie the impaired bone regeneration observed in aged periosteum.

### Age-induced degenerative changes in periosteal progenitor cells

**Fig. 3:**
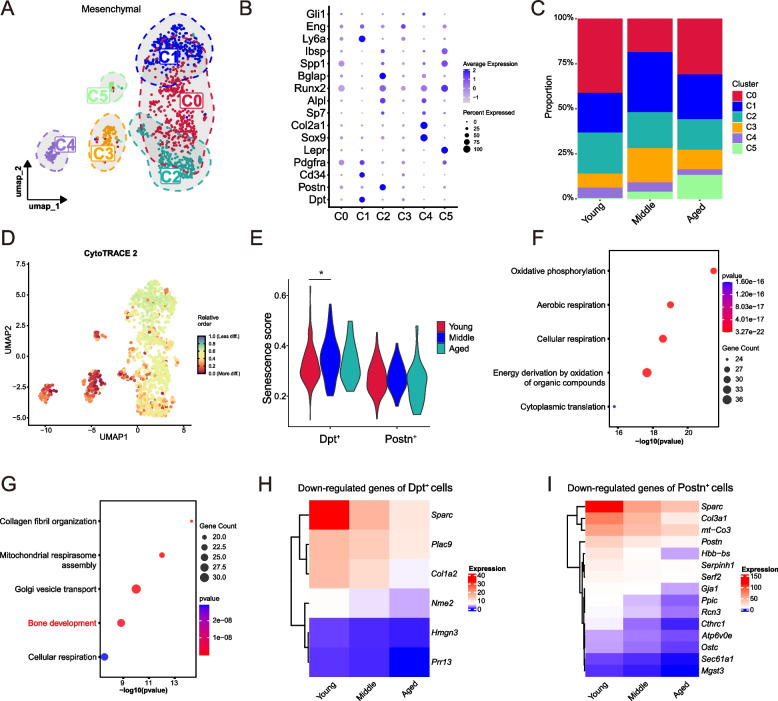
Distinct senescence responses in periosteal progenitor cells during aging. **A** UMAP plot illustrating the re-clustering results of progenitor cells. **B** Dot plots showing marker gene expression of periosteal fibrous and cambial layers. **C** Proportional distribution of progenitor cell subpopulations at different aging stages. **D** CytoTRACE2 plot showing the stemness of different progenitor subpopulations. **E** Senescence scores of each progenitor subpopulation using the SenMayo senescence gene set. **F** GO enrichment analysis of up-regulated genes in *Dpt*^+^ (C1) subpopulation between aged and young groups. **G** GO enrichment analysis of down-regulated genes in *Postn*^+^ (C2) subpopulation between aged and young groups. **H** Time-dependent expression levels of genes consistently downregulated in *Dpt*^+^ (C1) subpopulation. **I** Time-dependent expression levels of genes consistently downregulated in *Postn*^+^ (C2) subpopulation

The regenerative capacity of the periosteum relies on its resident osteogenic progenitor cells. Aging-induced downregulation of pathways such as oxidative phosphorylation, PI3K, IL-7 signaling, and extracellular matrix interaction suggests functional decline in mesenchymal cells (Fig. S3A). Previous studies have shown that osteogenic progenitor cells in the periosteum exhibit heterogeneity (Matthews et al. 2021; Xing et al. 2024). Clustering analysis identified six functionally distinct mesenchymal subpopulations from a total of 1162 Mesenchymal cells: C0 (high expressed *Runx2* and *Spp1*), C1 (high expressed *Dpt*), C2 (high expressed *Postn*), C3 (high expressed *Eng*), C4 (high expressed *Sox9*) and C5 (high expressed *Lepr*) (Fig. 3A, S3B, 3B), all marked by *Pdgfra*, a progenitor cell identity gene (Fig. 3B). The C0 and C2 subgroups are primarily associated with osteogenesis, while the C1 subgroup is linked to continuous collagen deposition. Additionally, C3 is associated with immune responses, C4 with chondrogenesis, and C5 with both classical and non-classical Wnt signaling pathways.

Prior research has pinpointed specific markers for periosteal stem cells across various anatomical layers (Liu et al. 2024). The *Dpt*⁺ (C1) progenitors reside in the fibrous layer and are key regulators of late-stage endochondral osteogenesis (Liu et al. 2024), while the *Postn*^+^ (C2) progenitors, located in the cambium layer, support bone homeostasis and early osteogenesis after fracture healing (Liu et al. 2024) (Fig. 3B, S3C). Aging leads to a significant reduction in *Postn*⁺ (C2) cells, whereas *Dpt*⁺ (C1) cell proportions remain stable (Fig. 3C). Stemness analysis indicates that *Dpt*⁺ (C1) progenitors exist in a relatively quiescent state compared to *Postn*⁺ (C2) progenitors (Fig. 3D). Although senescence scoring revealed a marked increase in *Dpt*⁺ (C1) progenitor senescence in middle age, it did not further escalate in aged group (Fig. 3E), potentially reflecting a loss of self-renewal due to impaired oxidative phosphorylation **(**Fig. 3F**)**. In contrast, *Postn*⁺ (C2) progenitors showed minimal senescence progression (Fig. 3E), yet aging was associated with reduced osteogenic gene expression in *Postn*⁺ (C2) progenitors, suggesting a potential decline in their osteogenic capacity (Fig. 3G).

We then identified genes consistently and significantly downregulated with aging in these periosteal progenitor subsets. Sparc, a key regulator of angiogenesis and osteogenesis, exhibited the most pronounced decline (Fig. 3H-I). In *Dpt*⁺ (C1) progenitors, *Nme2*, a stemness-related gene (Qi et al. 2021), and *Hmgn3*, linked to differentiation potential (Zhang et al. 2022), were markedly downregulated. Meanwhile, *Postn*⁺ (C2) progenitors showed consistent downregulation of osteogenic genes, including *Serpinh1* (Christiansen et al. 2010), Gja1 (Zhao et al. 2024), and *Cthrc1* (Chen et al. 2019), along with a significant reduction in *mt-Co3*, a crucial regulator of cellular energy metabolism. This age-driven transcriptional shift likely impairs bone homeostasis.

These findings indicate that aging induces significant alterations in periosteal progenitor cells, resulting in reduced osteogenic potential. Notably, the *Dpt*⁺ (C1) subpopulation is the primary periosteal osteogenic lineage to respond earliest to aging. These changes may contribute to the age-related deterioration of bone homeostasis. Although *Postn*⁺ (C2) periosteal progenitor cells exhibit relative resistance to age, their numbers and osteogenic capacity decline markedly with age, potentially due to limited energy supply. This depletion may contribute to age-associated disruptions in bone homeostasis and bone regeneration, including thinning of cortical bone and delayed healing. These findings highlight the importance of therapeutic strategies aimed at restoring the function of periosteal progenitor cells for treating age-related bone homeostasis disorders.

### Aging weakens the initial role of neutrophils in bone healing

**Fig. 4:**
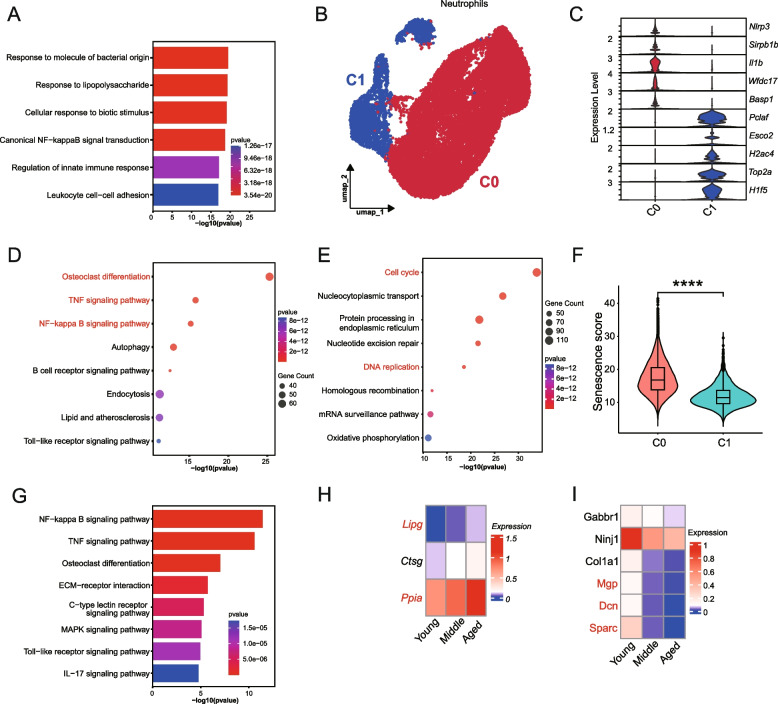
Neutrophil heterogeneity and functional changes during periosteal aging.** A** GO enrichment analysis of down-regulated DEGs in neutrophils between aged and young groups. **B** UMAP plot illustrating the re-clustering results of neutrophils. **C** Violin plots showing marker gene expression of C0-C1 neutrophil subpopulations. **D-E** KEGG enrichment analysis of genes specifically expressed in the (E) *Nlrp3*^*hi*^ (C0) and (F) *Pclaf *^*hi*^ (C1) neutrophils. **F** Senescence scores of *Nlrp3*^*hi*^ (C0) and *Pclaf *^*hi*^ (C1) neutrophils subpopulations, with *p*-values calculated using the Wilcoxon nonparametric test. **G** KEGG enrichment analysis of down-regulated genes in *Nlrp3*^*hi*^ (C0) neutrophils between aged and young groups. **H-I** Secreted proteins that are continuously (H) up-regulated and (I) down-regulated across different aging groups in *Nlrp3*^*hi*^ (C0) neutrophils

Neutrophils are among the first immune cells to respond to hematoma formation and play a critical role in the initiation of bone healing (Kovtun et al. 2016). To study transcriptional changes in neutrophils, DEGs were calculated during aging (Fig. S4A, Table S2). Compared to the young group, cellular respiration and energy supply were increased (Fig. S4B) and multiple pathways associated with the immune response were downregulated in the aged group (Fig. 4A). Given the heterogeneity, neutrophils were re-clustered to two subpopulations, C0 (*Nlrp3*^*hi*^) and C1 (*Pclaf*^*hi*^) (Fig. 4B). The *Nlrp3*^*hi*^ (C0) neutrophils expressed high levels of *Nlrp3* (Van Bruggen et al. 2023), *Il1b*, *Cxcl2* (De Filippo et al. 2013), key mediators of immune activation and recruitment (Fig. 4C). The *Nlrp3*^*hi*^ (C0) neutrophils were enriched in pathways related to inflammation and phagocytosis, as well as osteoclast differentiation (Fig. 4D). Conversely, the *Pclaf *^*hi*^ (C1) neutrophils were primarily associated with cell cycle and DNA replication pathways (Fig. 4E). Additionally, *Nlrp3*^hi^ neutrophils upregulated IL6-JAK-STAT3 and mTORC1 signaling, while TGFβ signaling was downregulated compared to *Pclaf*^*hi*^ neutrophils (Fig. S4C), suggesting a dominant role in immune response rather than proliferation. Moreover, age-related gene scoring revealed greater senescence in *Nlrp3*^*hi*^ (C0) neutrophils compared to the *Pclaf *^*hi*^ (C1) neutrophils (Fig. 4F). Focusing on the *Nlrp3*^*hi*^ neutrophils, we identified DEGs at different stages of aging (Fig. S4D, Table S3). The reduced signaling of NF-κB, TNF, Toll-like receptors, and IL-17 in aged *Nlrp3*^*hi*^ neutrophils in the aged group compared to the young group indicated a diminished capacity to activate and amplify inflammation, potentially impairing their ability to initiate bone healing (Fig. 4G). To assess the regulatory function of *Nlrp3*^*hi*^ neutrophils, we analyzed secreted proteins from DEGs consistently up- or downregulated with age **(**Fig. 4H-I**)**. Elevated *Lipg* expression may indicate increased neutrophil infiltration (Wang et al. 2023). However, factors related to bone formation and angiogenesis, such as Sparc (Jendraschak and Sage 1996; Rosset and Bradshaw 2016), Dcn (Adachi et al. 2022), and Mgp (Zhang et al. 2025), were significantly reduced in aged *Nlrp3*^*hi*^ neutrophils, suggesting a diminished contribution of the *Nlrp3*^*hi*^ neutrophils to bone regeneration with aging. These findings suggested that post-aging dysfunction of *Nlrp3*^*hi*^ neutrophils, with a diminished ability to activate and regulate inflammation, may hinder early effective inflammatory responses and delay bone healing.

### Aging alters the numbers and functions of macrophage subpopulations, impeding bone regeneration

Macrophages are crucial for bone repair and homeostasis, with their depletion significantly impairing healing (Vi et al. 2015). In aging macrophages, immune and inflammatory pathways were upregulated, whereas chondrogenic and osteogenic pathways were downregulated, indicating a decline in the supportive role of macrophages in osteogenesis with aging (Fig. S5A-B, 5A).

**Fig. 5:**
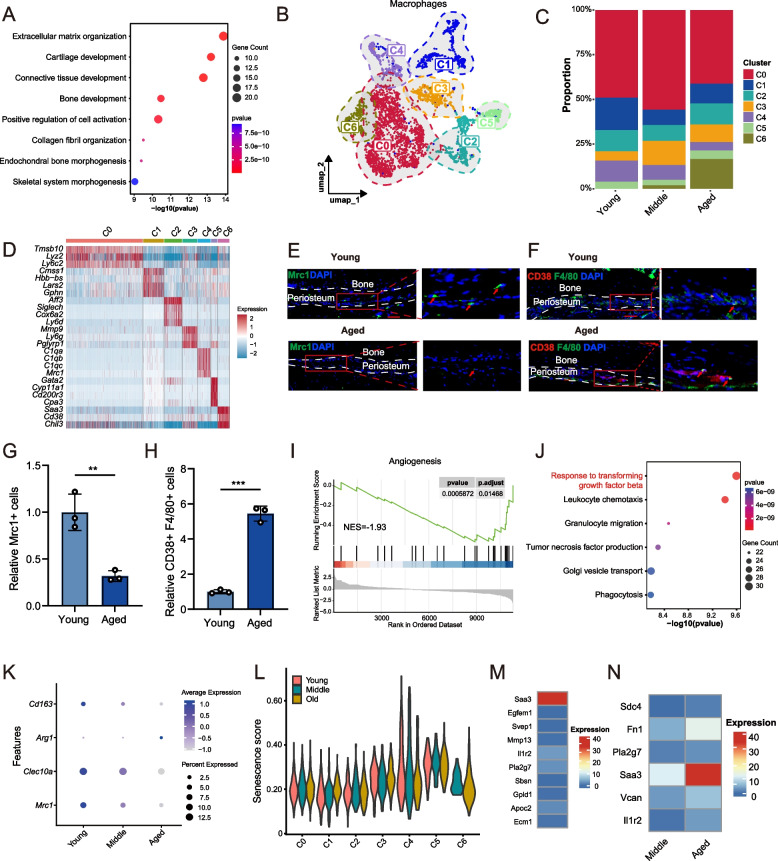
Macrophages composition changes during periosteal aging. **A** Gene Ontology (GO) enrichment analysis of genes down-regulated in macrophages between aged and young groups. **B** UMAP plot illustrating the re-clustering results of macrophages. **C** Variations in macrophage cell subtypes during aging. **D** Heatmap depicting marker genes expression across C0–C6 macrophage subpopulations. **E** Immunofluorescence staining of *Mrc1*⁺ (C4) macrophages in young and aged periosteum (tibia), with representative positive signals indicated by arrows (scale bar: 50 μm). The percentage of *Mrc1*⁺ cells in the aged periosteum is shown relative to that in the young periosteum. **F** Immunofluorescence staining of the *Cd38*^*hi*^ (C6) macrophages in young and aged periosteum (tibia), with representative positive signals indicated by arrows. Scale bar: 50μm. The percentage of *CD38*^+^ *F4/80*⁺ cells in the aged periosteum is shown relative to that in the young periosteum. **G** Quantification of immunofluorescence staining of *Mrc1*⁺ (C4) macrophages in the periosteum (tibia) of young and aged mice. The percentage of positive cells was counted and shown as a relative value to the young group. **H** Quantification of immunofluorescence staining of *Cd38*^*hi*^ (C6) macrophages in the periosteum (tibia) of young and aged mice. The percentage of positive cells was counted and shown as a relative value to the young group. **I** GSEA analysis revealing reduced enrichment of the angiogenesis signaling pathway in *Mrc1*^+^ macrophages in the aged group compared to the young group. **J** GO enrichment analysis of down-regulated DEGs in the *Mrc1*^+^ macrophages subpopulation between aged and young groups. **K** Age-dependent expression of M2 macrophage markers in the *Mrc1*^+^ (C4) macrophages. **L** Senescence scoring of macrophages subpopulations using the SenMayo senescence gene set. **M** Top 10 most abundantly expressed secretory factors in the *Cd38*^*hi*^ (C6) macrophages of aged periosteum. **N** Secretory factors that are upregulated in *Cd38*^*hi*^ (C6) macrophages following senescence compared to middle-aged group

Given the heterogeneity, we further re-clustered macrophages into seven subpopulations, from C0 to C6 (Fig. 5B-C). The function of each subpopulation was characterized by GO enrichment analysis (Fig. S5C). Notably, the *Mrc1*^+^ (C4) subpopulation declined with age, while the *Cd38*^hi^ (C6) subpopulation emerged predominantly in aged mice (Fig. 5C-D). These findings were confirmed by immunofluorescence staining (Fig. 5E-H). Both subpopulations expressed the common macrophage markers *F4/80* and *Cd68* (Fig. S5D). Although the *Mrc1*^+^ (C4) macrophages expressed pro-inflammatory markers, it also expressed M2 polarization markers such as *Cd163* (Barros et al. 2013), *Mrc1* (Martinez et al. 2008) and *Clec10a* (Tang et al. 2022), suggesting a mixed M1/M2 phenotype (Fig. S5D-E). In contrast, the *Cd38*^*hi*^ (C6) macrophages displayed minimal expression of classical M2 markers but high M1 markers, suggesting it is primarily M1-like (Fig. S5E).

These age-related shifts in the *Mrc1*^+^ and *Cd38*^*hi*^ macrophages warrant further investigations. GSEA revealed that *Mrc1*⁺ macrophages from aged periosteum exhibited reduced expression of secreted angiogenesis-related genes, suggesting a decline in their angiogenic signaling potential during aging (Fig. 5I), and the most significantly downregulated pathway was TGF-β signaling, a key regulator of M2 polarization and M1 inhibition (Fig. 5J). The *Mrc1*⁺ macrophages progressively lost M2 markers with age and exhibited higher senescence scores (Fig. 5K-L), whereas *Cd38*^*hi*^ macrophages showed lower senescence levels and upregulation of NF-κB signaling and oxidative phosphorylation (Fig. 5J, Fig. S5H). Notably, *Cd38*^*hi*^ macrophages highly expressed Saa3, an inhibitor of osteoblast differentiation and bone repair (Choudhary et al. 2018), with levels rising from middle-aged to aged groups (Fig. 5M-N). The origin of the increased *Cd38*^*hi*^ macrophages remains unclear; but their high expression of circulating monocyte marker genes (*Cxcr2* (Wang et al. 2018) and *Ccr2* (Krenkel et al. 2018)), and minimal expression of the resident macrophage marker genes (*Cx3cr1* (Koscsó et al. 2020)) suggests a probable circulatory origin (Fig. S5I). In conclusion, aged macrophages exhibit heightened inflammation and diminished osteogenic support. Aging leads to a decrease in the number of *Mrc1*^+^ macrophages with mixed M1/M2 phenotypes and a decrease in the M2 phenotype. In contrast, the M1-like *Cd38*^*hi*^ macrophages increased and exhibited elevated expression of the bone formation-inhibitory molecule Saa3. These senescent-induced alteration is detrimental to bone physiological homeostasis and healing after injury.

### Aging-driven crosstalk between progenitor and immune cells impairs bone homeostasis and regeneration

**Fig. 6:**
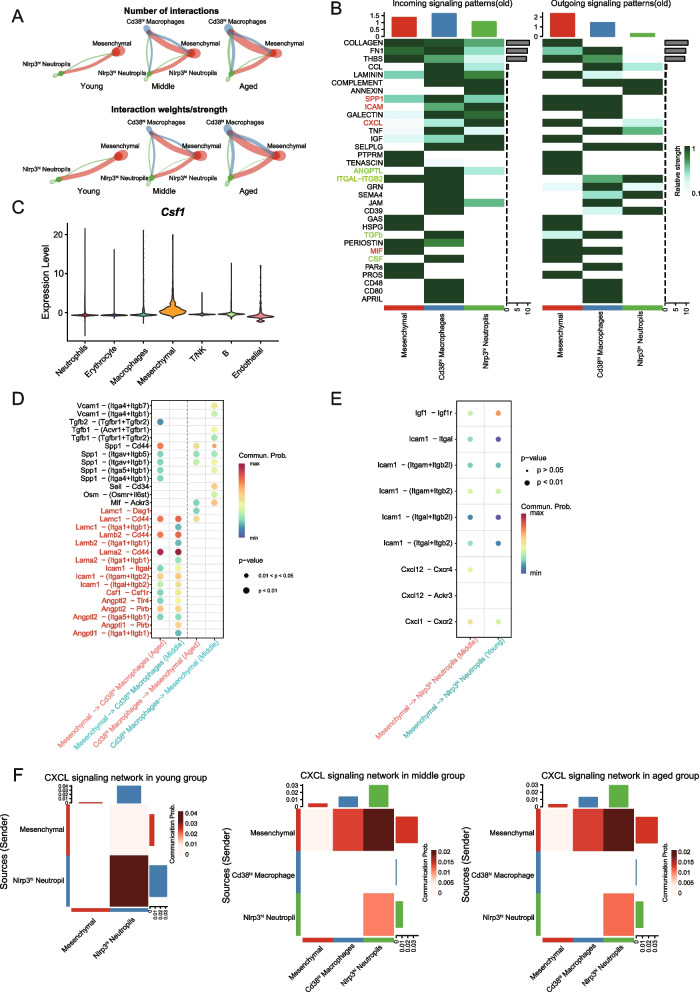
Age-induced changes in communication between progenitor cells, neutrophils and macrophages. **A** Age-dependent changes in the number and strength of communications between progenitor cells, *Cd38*^*hi*^ macrophages, and *Nlrp3*^*hi*^ neutrophils, which are significantly increased with aging. **B** Patterns of signal incoming and outgoing between progenitor cells, *Cd38*^*hi*^ macrophages, and *Nlrp3*^*hi*^ neutrophils in the aged group. **C** The expression levels of *Csf1* in different cell subpopulations of the periosteum. **D** Ligand-receptor pairs between progenitor cells and *Cd38*^*hi*^ macrophages in middle-aged and aged groups. **E** Ligand-receptor pairs between progenitor cells and *Nlrp3*^*hi*^ neutrophils in young and middle-aged groups. **F** Age-dependent changes in CXCL signaling between progenitor cells, *Cd38*^*hi*^ macrophages, and *Nlrp3*^*hi*^ neutrophils

After bone injury, hematoma formation is an early event in the healing process, with neutrophils being the first to be activated, followed by macrophages (Kovtun et al. 2016). Notably, the primary regulatory signals that influence macrophages and neutrophils in the periosteum originate from osteogenesis-associated progenitor cells, increased significantly with aging, rather than from other immune cells (Fig. 2A). Moreover, the most significantly altered subpopulations in macrophages and neutrophils were M1-like *Cd38*^*hi*^ macrophages, and aging-induced dysfunctional *Nlrp3*^*hi*^ neutrophils, respectively. Furthermore, we downloaded scRNA-seq data from the fracture callus of young and aged mice (GSE198666) and performed subclustering of macrophages and neutrophils. We found a significant increase in the proportions of *Cd38*⁺ macrophages and *Nlrp3*⁺ neutrophils in aged mice compared to young controls. These results are consistent with our observations in the periosteum of aged mice and further support the robustness of our analysis (Fig. S6A). Thus, we next examined the link between these immune cells and periosteal progenitor cells during aging. Signals from progenitor cells increase with aging (Fig. 6A). To further explore age-related alterations in communication between progenitor cells and the *Cd38*^*hi*^ macrophages and *Nlrp3*^*hi*^ neutrophils, both outgoing and incoming signaling patterns were analyzed (Fig. S6B-C, 6B). Progenitor cells were found to regulate macrophages predominantly through LAMININ, ANGPTL, and CSF signaling pathways, with CSF signaling playing a critical role in macrophages proliferation and differentiation. Notably, Csf1 secreted by progenitor cells was minimal at young age but increased substantially after middle age, primarily driven by the C1, C3, and C5 progenitor cells (Fig. S6D). Analysis of *Csf1* expression across all populations confirmed that progenitor cells represented a primary source of CSF signaling (Fig. 6C). Most key regulatory signals from the *Cd38*^*hi*^ macrophages to progenitor cells are significantly attenuated with aging, including TGFβ, OSM, SELL, MIF, and SPP1, which are involved in regulating the balance of bone formation and bone resorption (Ando et al. 2024; Chen et al. 2012; Faqeer et al. 2023; Onodera et al. 2006; Thankamony and Sackstein, 2011; Zheng et al. 2019) (Fig. 6D). To investigate the influence of young and aged pSSCs on macrophages, we performed co-culture experiments using macrophages and pSSCs derived from either young or aged mice. The results showed that, compared to young pSSCs, aged pSSCs significantly enhanced macrophage migration (Fig. S6E-F).

The signaling intensity by which progenitor cells modulated *Nlrp3*^*hi*^ neutrophils also underwent alterations with aging, including IGF, CXCL, and ICAM (Fig. 6E). CXCL signaling, a classical chemotactic signal, is mediated primarily by CXCL1 and CXCL12 in the progenitor-neutrophil communication during young age. However, with the progression of aging, the intensity of CXCL signaling increased significantly (Fig. 6E-F), and the fibrous layer SSCs (C1) contributed more to CXCL1 and CXCL12 secretion compared to the cambium layer SSCs (C0) (Fig. S6G).

In conclusion, the above findings suggest that during aging, progenitor cells recruit more *Cd38*^*hi*^ macrophages and *Nlrp3*^*hi*^ neutrophils through CSF and CXCL signaling respectively. This significant alteration in cellular communication may further impair periosteum-mediated bone regeneration.

## Discussion

Aging is known to impair bone regenerative capacity. This study offers new insights into the molecular mechanisms behind this decline, with a focus on cellular function and immune-mesenchymal interactions. Through a comprehensive single-cell transcriptomic analysis of periosteal aging, we reveal significant disruptions in bone homeostasis. Aging resulted in a decline in periosteal mesenchymal progenitors, an increased senescence burden, and impaired osteogenic potential, which contributed to the destabilization of periosteal integrity. Specifically, *Postn*⁺ periosteal progenitors, which are essential for periosteal osteogenesis and cortical bone repair (Perrin and Colnot 2022), exhibited transcriptional signatures indicative of diminished differentiation potential. Meanwhile, *Dpt*⁺ progenitors, recently identified as critical for fracture healing (Matsushita et al. 2020), displayed early aging signatures, suggesting an intrinsic aging-related impairment that compromises bone regeneration. The depletion and dysfunction of these progenitors align with previous studies showing that mesenchymal stem cell (MSC) senescence and reduced osteogenic activity contribute to age-related bone loss (Pignolo et al. 2021).

A key feature of aged periosteum was the shift in immune cell composition and signaling, characterized by increased infiltration of *Cd38*^hi^ macrophages and *Nlrp3*^hi^ neutrophils, both of which are associated with chronic inflammation and impaired osteogenesis (Okamoto et al. 2017). Additionally, previous studies have reported that macrophages are a major driver of impaired healing during aging, further supporting our findings (Vi et al. 2018).

Aged mesenchymal progenitors upregulated CSF1 and CXCL chemokine signaling, potentially promoting the recruitment of inflammatory monocytes and neutrophils, thereby amplifying a pro-inflammatory environment. This is consistent with the concept of inflammaging, where chronic low-grade inflammation disrupts tissue homeostasis and promotes age-related pathologies, including osteoporosis (He et al. 2023).

Additionally, aged mesenchymal progenitors exhibited upregulation of SASP factors, such as IL-6, CCL2, and TNF-α, which are known to drive osteoclastogenesis and inhibit osteoblast differentiation (Kirkland and Tchkonia 2017). This inflammatory shift disrupts the finely tuned balance between bone formation and resorption, leading to increased bone fragility. Prior studies have demonstrated that inflammatory cytokines such as TNF-α and IL-1β can impair osteoblast function and promote osteoclast-mediated bone resorption, accelerating age-related bone loss (Ginaldi et al. 2005).

Our study also highlights the age-related decline in periosteal regeneration, marked by impaired intercellular communication. The suppression of pro-osteogenic pathways, such as EGF and PTN signaling, alongside the upregulation of inflammatory cytokine-mediated interactions (CSF1, TNF, and CCL signaling), reflects a shift toward a bone-resorption-favoring microenvironment. This aligns with previous findings that aging disrupts skeletal stem cell (SSC) niches, impairing their ability to respond to injury and regenerate bone (Ambrosi et al. 2021).

The observed changes in mesenchymal progenitors, immune cells, and vascular components contribute to the broader phenomenon of bone homeostasis dysregulation, a hallmark of osteoporosis and delayed fracture healing in the elderly. The increased pro-inflammatory macrophages and neutrophils may exacerbate bone loss by enhancing osteoclast activation, as observed in osteoporosis models (Siddiqui and Partridge 2016). Additionally, the age-related decline in periosteal progenitors mirrors findings in skeletal aging studies, where diminished stem cell function leads to impaired fracture healing (Chan et al. 2015).

Given these findings, targeting inflammatory signaling pathways or rejuvenating periosteal progenitors may represent promising therapeutic strategies. Modulating CSF1, TNF, or CXCL signaling could counteract the age-related pro-inflammatory shift, restoring bone homeostasis and mitigating osteoporotic fracture risk. Additionally, interventions aimed at enhancing vascular function, such as pro-angiogenic therapies or exercise-induced vascular remodeling, may improve periosteal regenerative capacity in aging populations (Willam et al. 2002).

However, our study also has several limitations. While some senescence-associated features were detected, their overall dynamics across age groups were modest, and not all cell populations exhibited a clear progression from middle-aged to aged mice. This suggests that cellular senescence in the periosteum may be heterogeneous and highly context-dependent. In addition, technical limitations such as the relatively low abundance of certain cell types, including mesenchymal cells, may have limited our ability to detect subtle changes. Future studies with lineage-specific enrichment or spatial transcriptomic approaches may help to better resolve these aspects. Although we performed additional validation experiments, including flow cytometry and co-culture migration assays, several key conclusions of this study remain primarily inferred from transcriptomic analyses. Future studies incorporating lineage tracing, functional perturbation, and injury models will be necessary to further substantiate the mechanistic roles of these cell populations in periosteal aging and bone regeneration.

In summary, our study reveals that periosteal aging is driven by a complex interplay between mesenchymal progenitor senescence, chronic inflammation, and vascular dysfunction, leading to bone homeostasis disruption. These findings provide novel insights into the cellular and molecular mechanisms underlying skeletal aging and may guide future strategies to preserve bone health in the elderly.

## Methods

### Acquisition of animals and long bones

Long bones were harvested from wild-type C57BL/6 J mice across three age groups: 3 months, 9 months, and 18 months, with six males per group (Aniphe Biolaboratory Inc.). The acquisition protocol was as follows: We performed cardiac perfusion with PBS prior to periosteum harvesting in all animals. Following cervical dislocation for euthanasia, the animals were sterilized by external application of 75% ethanol. The lower limbs were then aseptically dissected, and the overlying skin, muscles, fat, and other soft tissues were meticulously removed. The intact femur and tibia were subsequently isolated for further processing.

### Periosteal cell dissociation

The periosteal tissue was carefully dissected from the femora and tibiae of mice under a stereomicroscope to ensure precise separation of the periosteum from underlying bone and surrounding soft tissues. To address these issues and minimize inter-individual variability, we carefully designed our experiments by pooling periosteal tissues from six mice for each age group. To minimize batch effects and ensure data comparability, tissue digestion, cell isolation, and subsequent procedures for all experimental groups were performed in parallel. The periosteal tissue was dissociated using a mixed enzyme solution prepared according to the manufacturer’s protocol (Majorbio Bio-Pharm Technology Co. Ltd., Shanghai, China). The solution contained 2 mg/ml type II collagenase, 0.5 mg/ml dispase, 50 U/ml DNase. After preheating the mixed enzyme solution at 37 °C for 30 min, periosteal tissue was incubated with the enzyme mixture in a water bath at 37 °C. The digestion process was repeated four times, each for 15 min. After each digestion, the suspension was filtered through a 40 μm mesh and neutralized with an equal volume of complete medium (α-MEM containing 10% fetal bovine serum and 1% penicillin–streptomycin). The remaining tissue was subjected to a second round of digestion. At the end of the four digestions, the cell suspension was centrifuged at 300 g for 5 min. The pellet was resuspended in 1 mL of 1 × erythrocyte lysis buffer for 5 min, washed with 10 mL of ice-cold PBS, and centrifuged again at 300 g for 5 min. The final cell pellet was resuspended in 50 μL of PBS. Viability was assessed using the AO/PI staining method and an automated cell counter, with cell viability confirmed to be > 80%. The concentration of the cell suspension was adjusted to approximately 1000 cells/μL.

### Macrophage–periosteal stem cell co-culture and migration assay

To investigate the influence of pSSCs from different age groups on macrophage behavior, we performed co-culture experiments using pSSCs isolated from young (3-month-old) and aged (18-month-old) C57BL/6 mice. pSSCs were isolated and cultured under standard conditions until reaching approximately 80% confluence. Bone marrow–derived macrophages were harvested from wild-type mice and seeded into Transwell inserts (8-μm pore size) placed above the pSSCs monolayers. After 24 h of co-culture, macrophage migration through the membrane was assessed by crystal violet staining and quantified under a microscope. The results demonstrated that aged pSSCs significantly promoted macrophage migration compared to their young counterparts.

### Flow cytometric analysis of neutrophils and macrophages in periosteal tissue

To assess the proportions of neutrophils and macrophages in young and aged periosteum, we performed flow cytometric analysis on periosteal tissues harvested from young (3-month-old) and aged (18-month-old) mice. The periosteal tissues were carefully dissected from tibia and femur, and enzymatically digested into single-cell suspensions using a collagenase-based digestion protocol. For neutrophil analysis, cells were stained with anti-CD45 and anti-Ly6G monoclonal antibodies. Neutrophils were defined as CD45⁺Ly6G⁺ cells. For macrophage analysis, cells were stained with anti-CD45 and anti-F4/80 monoclonal antibodies, and macrophages were identified as CD45⁺F4/80⁺ cells. The percentages of neutrophils and macrophages were calculated as a proportion of total CD45⁺ immune cells in each sample.

### Single-cell RNA-seq library preparation and sequencing

Library preparation and sequencing were conducted by Majorbio Bio-Pharm Technology Co. Single-cell suspensions were loaded onto the Chromium Single Cell Controller (10 × Genomics) to generate single-cell gel bead emulsions, following the manufacturer’s guidelines (Chromium Single Cell 3ʹ v3.1). The Chromium Single-Cell 3' Gel Bead and Library V3 Kit was used for cDNA synthesis and amplification, and sequencing was carried out on an Illumina NovaSeq 6000 platform.

### Fastq file processing, quality control, and preliminary analysis

The raw sequencing data were processed using Cell Ranger software (version 6.1.1) to generate gene expression matrices based on the mm10 genome assembly. The pre-processed mouse reference genome (mm10) was downloaded from the 10 × Genomics website. The gene expression matrices were imported into R for downstream analysis using the Read10X function in Seurat (version 4.1.1). Cells with fewer than 200 genes or more than 15% mitochondrial gene content were excluded from further analysis. DoubletFinder package (version 2.0.4) was used to computationally identify and exclude potential doublets. Data normalization and scaling were performed using the “SCTransform” function. Data integration was achieved using “PrepSCTIntegration”, “FindIntegrationAnchors”, and “IntegrateData” functions. Principal component analysis (PCA) was performed on the integrated dataset using “ScaleData” and “RunPCA”, followed by clustering using the “FindNeighbors” and “FindClusters” functions. Dimensionality reduction was visualized with UMAP using the “RunUMAP” function. DEGs for each cluster were identified using the “FindMarkers” function in Seurat, with the following filtering criteria: avg_log2FC > 0.25 and p_val < 0.05. The identified DEGs were cross-referenced with the CellMarker database and previous literature to assign cell types to each cluster.

### Enrichment analysis

KEGG pathway analysis and Gene Ontology (GO) annotation were performed using the R package clusterProfiler (version 4.0). Gene Set Enrichment Analysis (GSEA) was performed using the Hallmark gene set from the MSigDB database. For all enrichment analyses, only DEGs with p.adjust < 0.05 were included. If the number of DEGs with p.adjust < 0.05 was insufficient for meaningful enrichment analysis, the filter was relaxed to p_val < 0.05.

### Trajectory Analysis and Stemness analysis

Trajectory analysis was performed using the Slingshot package (version 2.14.0) to infer potential transitions between cell subpopulations. UMAP was used for dimensionality reduction prior to trajectory analysis. The "slingshot" function was employed to model the smooth progression of cells through different stages (Street et al. 2018). The CytoTRACE package was utilized to obtain a CytoTRACE score for each subpopulation, allowing us to infer their respective differentiation status (Gulati et al. 2020).

### Cell-to-cell communication analysis

Cell-to-cell communication was analyzed using CellChat (version 1.6.1), a package that estimates the likelihood of intercellular communication by integrating user-provided gene expression data with known interactions between signaling ligands, receptors, and their cofactors. Communications involving fewer than 10 cells were excluded from the analysis. In addition to assessing communication within individual age groups, the “mergeCellChat” function was employed to integrate datasets from two age groups. This allowed for the synchronization and comparison of cell–cell communication patterns, signaling alterations, and receptor-ligand pair variations across different age cohorts.

### Senescence scoring

Senescence scores were calculated using the SenMayo gene set (PMID: 35,974,106) (Saul et al. 2022), with the “AddModuleScore” function in Seurat. The SenMayo gene set was originally defined as a panel of 125 senescence-associated genes through an extensive literature-based approach and was subsequently validated across multiple datasets from aged human and mouse tissues, including those demonstrating changes after senescent cell clearance (Saul et al. 2022). In cases where non-expressed genes led to errors during scoring, these genes were excluded from the set before recalculating the score. Statistical significance of the senescence scores was assessed using non- Wilcoxon tests from the ggpubr package.

### Immunofluorescence staining

After obtaining the tissues, they were fixed in PFA for 1 day and decalcified in 14% EDTA for 28 days after rinsing, with a fluid change once every 2–3 days. after dehydration in 30% sucrose overnight, samples were embedded using OCT; after that, 15µm-thick frozen sections were performed and preserved at −80° C. The samples were then incubated for 1 h at room temperature (RT) with 5% goat serum in PBS. For immunofluorescence staining, samples were blocked with 5% goat serum in PBS for 1 h at RT followed by incubation with primary antibody (1:200, Supplementary Table 7) at 4 °C overnight. The secondary antibody (1:200) was conjugated to anti-rabbit Alexa Fluor®594 or anti-mouse Alexa Fluor®488 and incubated for 2 h at RT. Sections were blocked using a DAPI-containing blocking agent. All sections were examined under a Zeiss 900 confocal microscope (Zeiss, Thornwood, NY).

### β-gal staining

Frozen sections of periosteum from young (3 months) and aged (18 months) mice were prepared. After 10 min at room temperature, the sections were washed three times with water (5 min each). They were then fixed with β-gal staining fixative for 5 min, followed by three washes with water (5 min each). The staining solution was prepared according to the manufacturer's instructions, applied to the tissue, and incubated overnight at 37 °C. After washing with water three times, the sections underwent dehydration through a graded alcohol series and were cleared with xylene. Finally, the sections were mounted with neutral resin.

### Statistical analysis

All statistical analyses and graphical representations were performed in R (version 4.1.1) using R package ggpubr. The *p*-value was calculated using Wilcoxon test. *P < 0.05 and **P < 0.01 were considered significant.

## Supplementary Information

Supplementary Material 1. Supplementary Figures. Fig. S1. Transcriptional characteristics of cell subpopulations in periosteum. Fig. S2. Alterations in cell communication patterns between young and aged periosteum. Fig. S3. Enrichment analysis of age-associated changes in periosteal progenitor cell populations. Fig. S4. Gene enrichment characteristics of neutrophils. Fig. S5. Characterization of macrophage subpopulations. Fig. S6. Key signals mediating changes in progenitor-immune cell crosstalk.

Supplementary Material 2. Supplementary Tables. Table S1. DEGs between aged and young groups. Table S2. DEGs in neutrophils between aged and the young groups. Table S3. DEGs in Nlrp3hi(C0) neutrophils between aged and the young groups.

## Data Availability

The scRNA-seq data generated in this study are publicly available through the Gene Expression Omnibus (GEO) with the accession number GSE297256. All other data are available from the corresponding author on request.

## Abbreviations

pSSCs: Periosteal skeletal stem cells
β-gal: Beta-galactosidase
DEG: Differentially expressed genes
MSC: Mesenchymal stem cell
SSC: Skeletal stem cell
