# Supplementary material for: Single-cell mapping reveals age-related alterations in periosteal progenitor cells and immune microenvironment
Source: Cell Regen. 2025 Nov 17;14:46. doi: 10.1186/s13619-025-00263-9 (PMC12623539; doi:10.1186/s13619-025-00263-9)
Supplement: Supplementary file 1 — Supplementary Material 1. Supplementary Figures. Fig. S1. Transcriptional characteristics of cell subpopulations in periosteum. Fig. S2. Alterations in cell communication patterns between young and aged periosteum. Fig. S3. Enrichment analysis of age-associated changes in periosteal progenitor cell populations. Fig. S4. Gene enrichment characteristics of neutrophils. Fig. S5. Characterization of macrophage subpopulations. Fig. S6. Key signals mediating changes in progenitor-immune cell crosstalk. [file 13619_2025_263_MOESM1_ESM.docx]

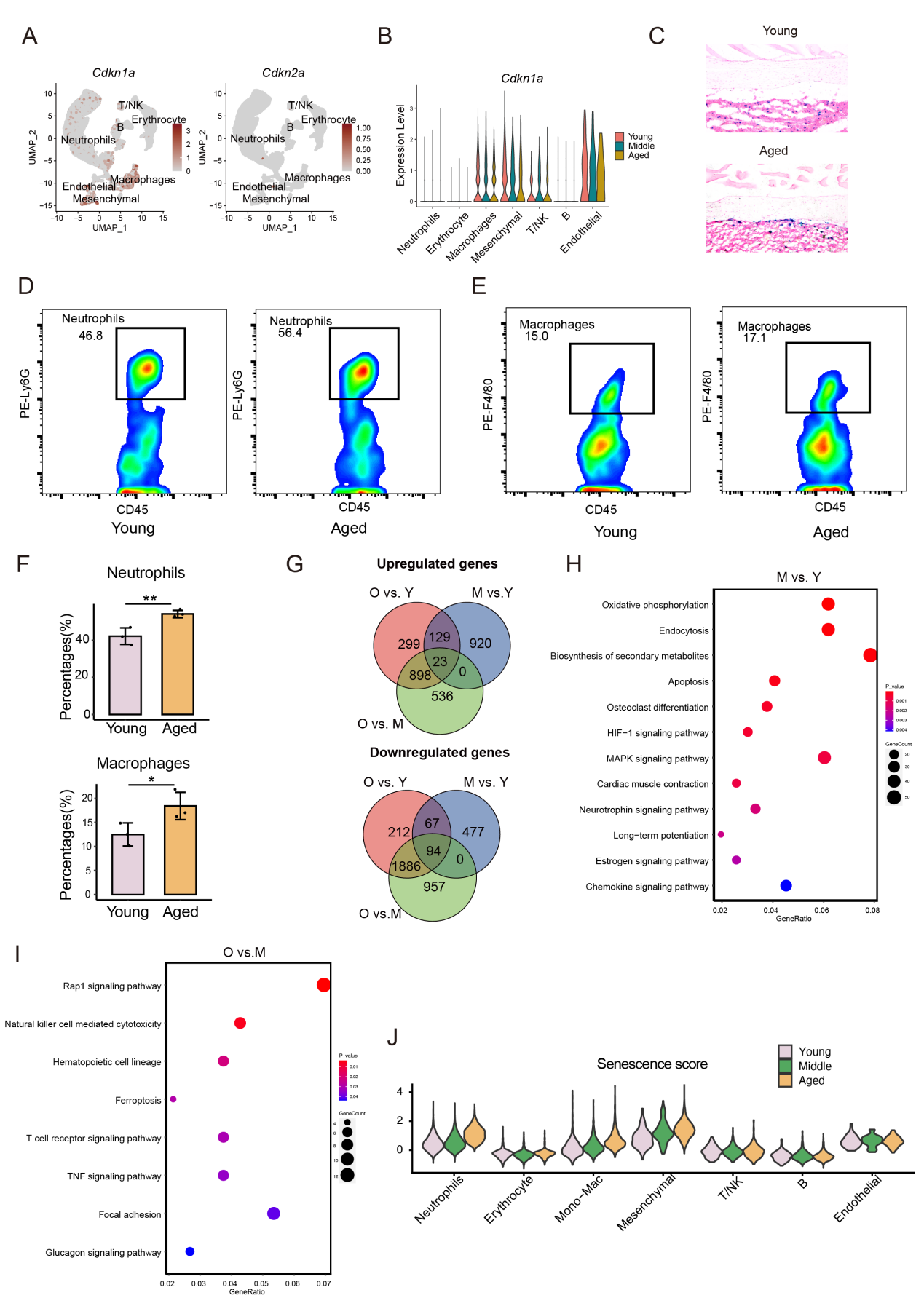


**Supplementary Figure 1. Transcriptional characteristics of cell subpopulations in periosteum**

**A**. Expression patterns of the senescence markers p21 and p16 in the periosteum across different cell subpopulations.

**B**. Age-related expression changes of p21 in different cell subpopulations of the

periosteum.

**C**. β-gal staining of young and aged periosteum.

**D**. Representative images of flow cytometry analysis of neutrophils in young and aged periosteum.

**E**. Representative images of flow cytometry analysis of macrophages in young and aged periosteum.

**F**. Quantification of flow cytometry analysis of neutrophils and macrophages (n = 3).

**G**. Up-regulated or down-regulated DEGs between different time points of aging (O vs. Y: Aged vs Young, O vs. M: Aged vs Middle, M vs. Y: Middle vs Young

**H**. Signaling pathways specifically upregulated in the periosteum of middle-aged mice compared to young mice.

**I**. Signaling pathways specifically upregulated in the periosteum of aged mice compared to middle-aged mice.

**J**. The senescence scores for each cell subpopulation across the young, middle-aged, and aged groups.


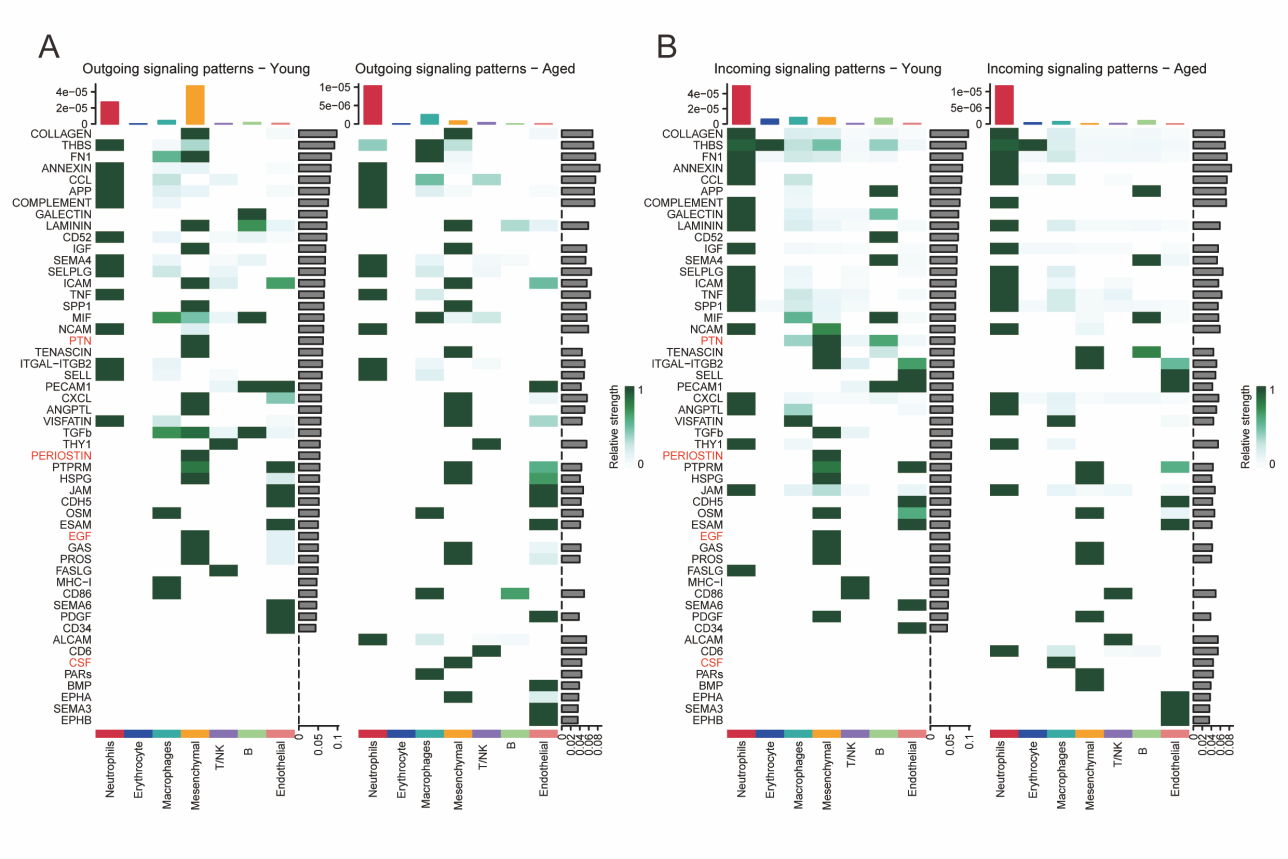


**Supplementary Figure 2. Alterations in cell communication patterns between**

**young and aged periosteum**

**A**. A comparison of outgoing signaling patterns among individual subpopulations of the periosteum between young and aged groups.

**B**. A comparison of incoming signaling patterns among individual subpopulations of

the periosteum between young and aged groups.


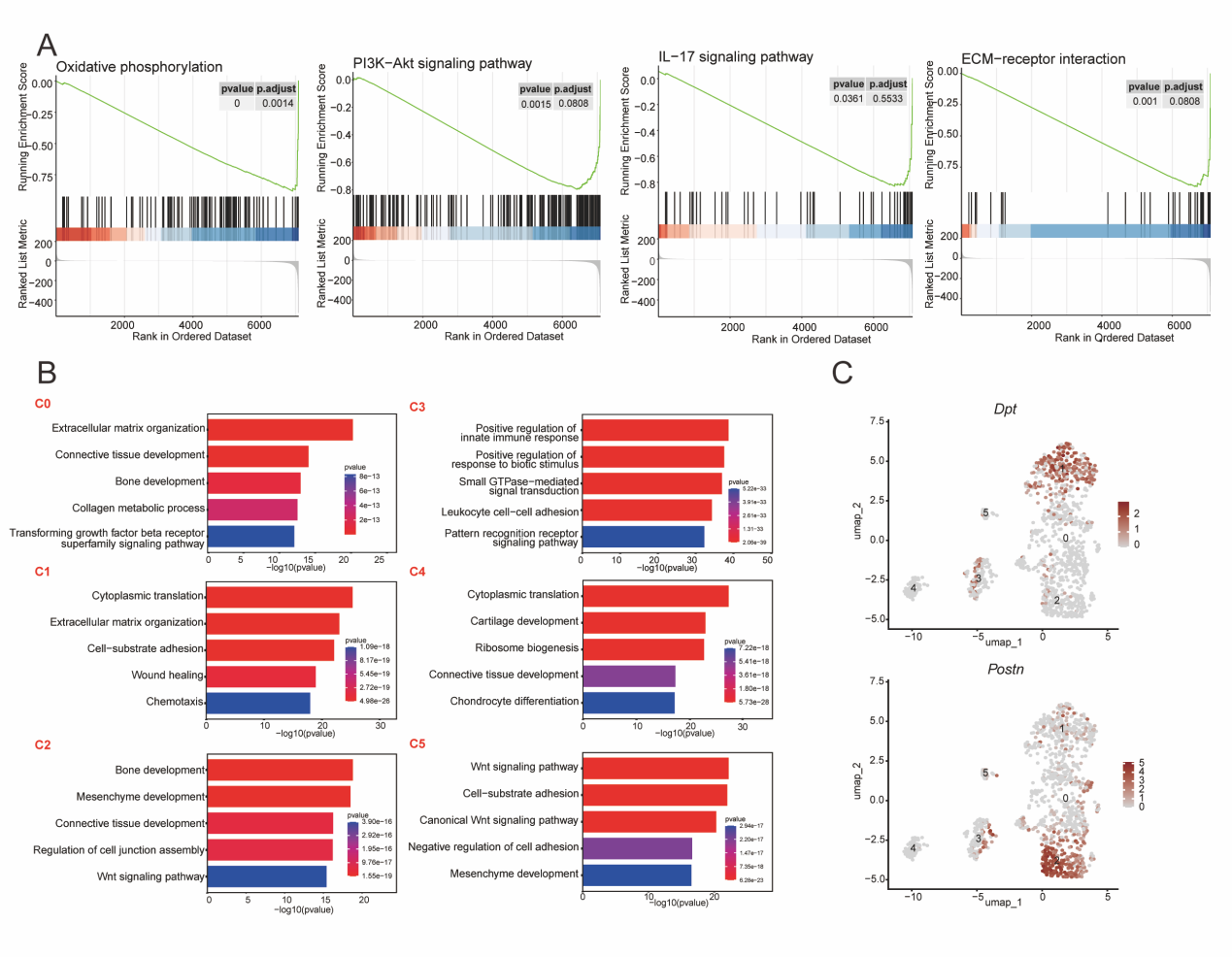


**Supplementary Figure 3. Enrichment analysis of age-associated changes in periosteal progenitor cell populations**

**A**. GSEA analysis revealing the enrichment of the Oxidative phosphorylation，

PI3K−Akt signaling pathway, IL−17 signaling pathway and ECM−receptor

interaction in progenitor cells in aged group compared to young group.

**B**. GO enrichment analysis of marker genes in different cell subpopulations of the

periosteal progenitor.

**C**. The expression of *Dpt* and *Postn* in periosteal progenitor.


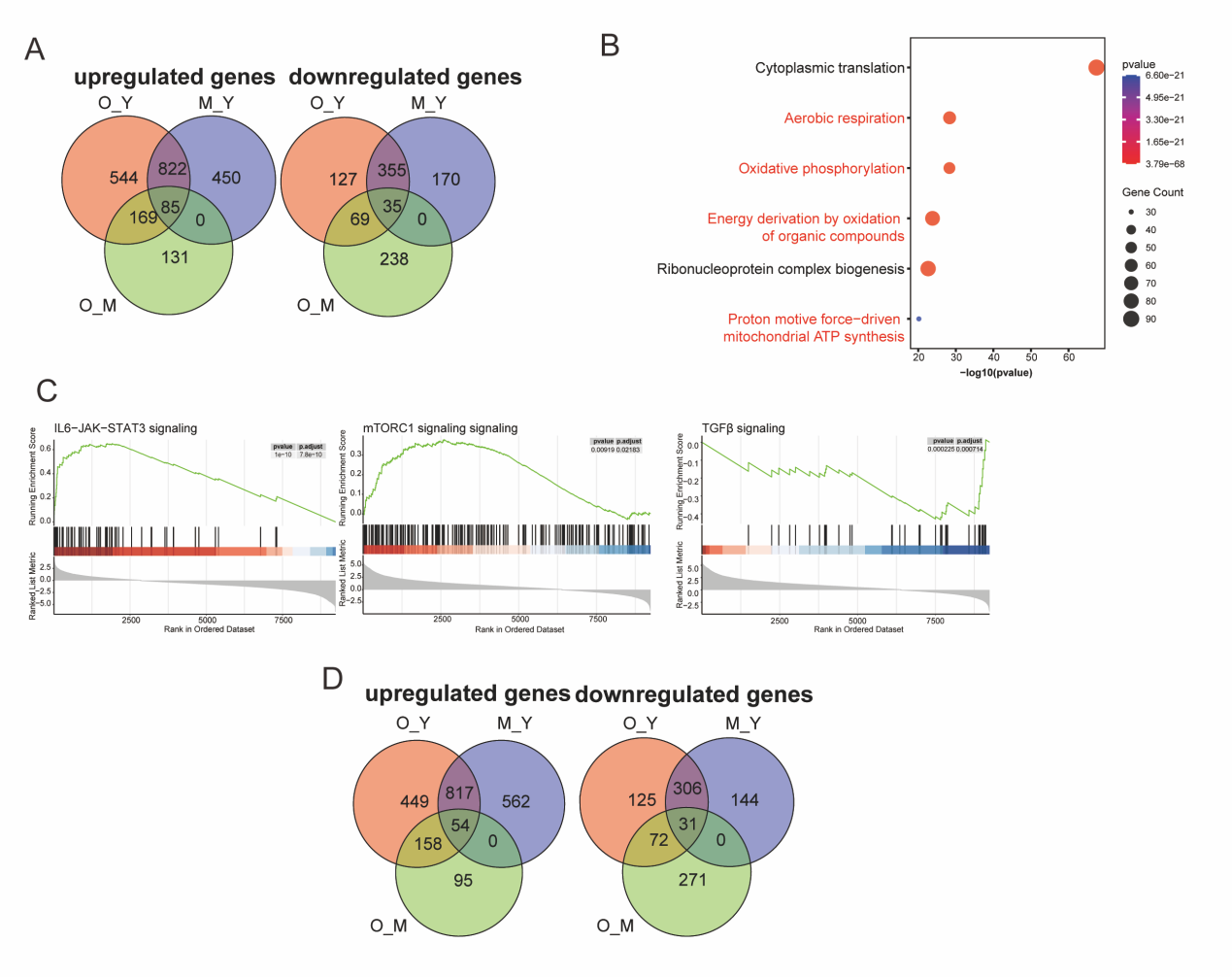


**Supplementary Figure 4. Gene enrichment characteristics of neutrophils**

**A**. Up-regulated or down-regulated DEGs in neutrophil subpopulations during periosteal aging (O vs. Y: Aged vs Young, O vs. M: Aged vs Middle, M vs. Y: Middle vs Young.

**B**. GO enrichment analysis of up-regulated DEGs between aged and young groups.

**C**. GSEA enrichment analysis of DEGs in the *Nlrp3*^hi^ (C0) subpopulation compared to *Pclaf* ^hi^ (C1) subpopulation.

**D**. Up-regulated or down-regulated DEGs between different time points of aging *Nlrp3*^hi^ (C0) neutrophils.


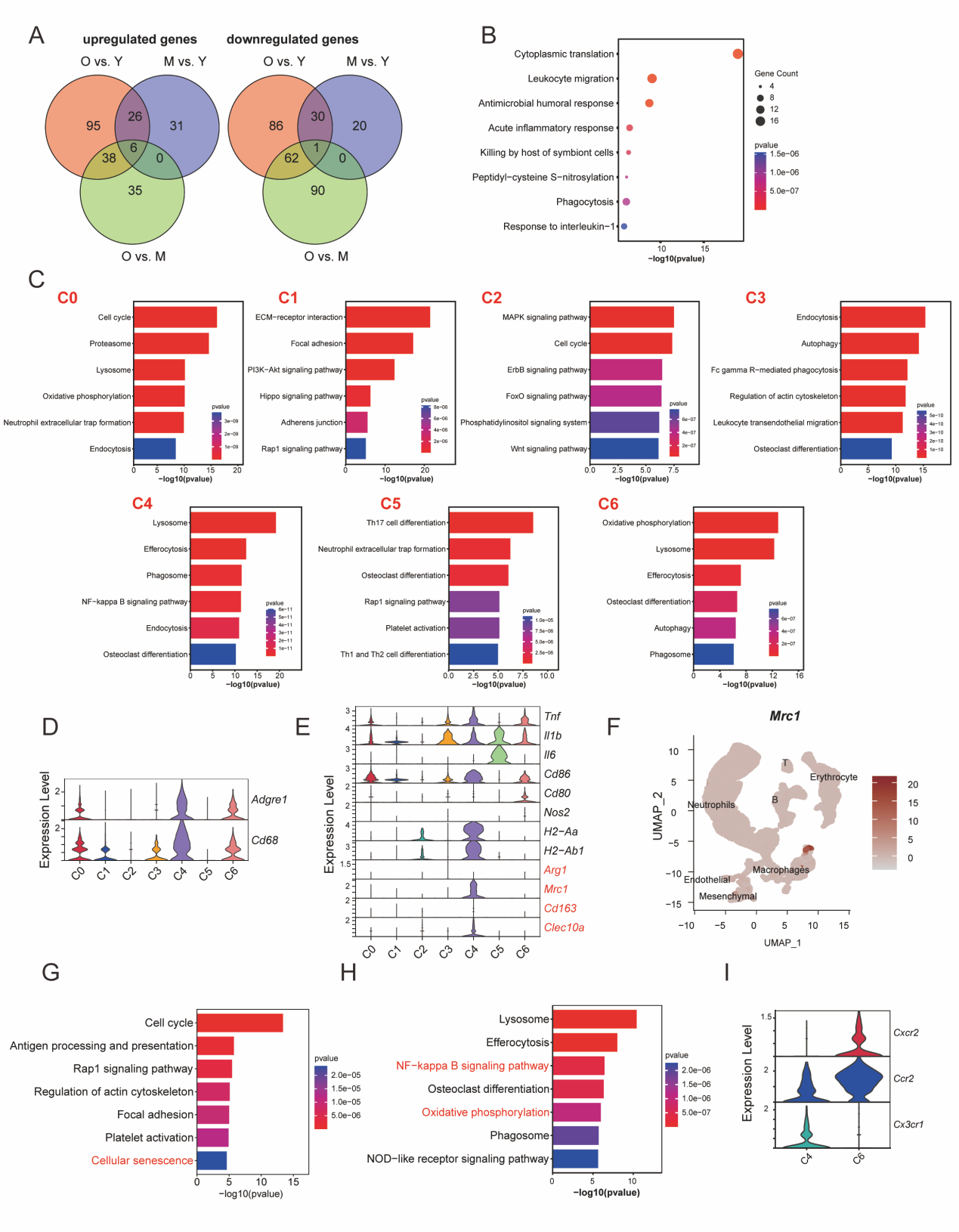


**Supplementary Figure 5. Characterization of macrophage subpopulations**

**A**. Up-regulated or down-regulated DEGs between different aging stages of macrophages (O vs. Y: Aged vs Young, O vs. M: Aged vs Middle, M vs. Y: Middle vs Young).

**B**. GO enrichment analysis of DEGs between aged and young groups.

**C**. KEGG enrichment analysis of highly expressed marker genes in each monocyte

macrophage subpopulation.

**D**. Violin plots showing the expression of macrophage markers.

**E**. Expression of markers associated with macrophage polarization and function in

monocyte-macrophage subpopulations.

**F**. Expression levels and distribution of *Mrc1* (*Cd206*) across all periosteal cells.

**G**. KEGG enrichment analysis of genes down-regulated in the *Cd38*^hi^ (C6)

macrophages compared to other subpopulations in the aged periosteum.

**H**. KEGG enrichment analysis of genes up-regulated in the *Cd38*^hi^ (C6) macrophages

compared to other subpopulations in the aged periosteum.

**I**. Expression levels for circulating and resident macrophages markers.


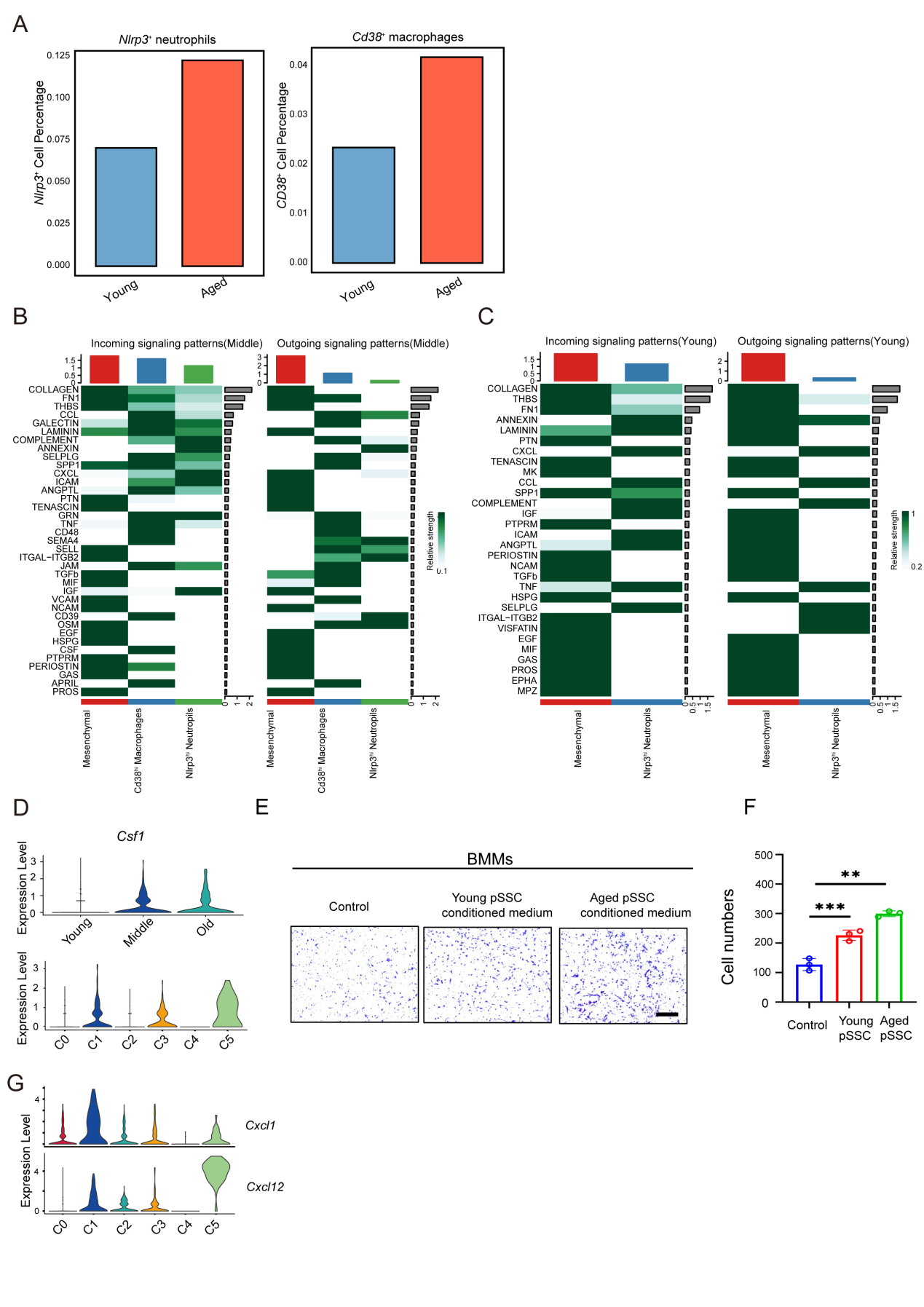


**Supplementary Figure 6. Key signals mediating changes in progenitor-immune**

**cell crosstalk**

**A.** Proportions of *Nlrp3*^hi^ neutrophils and *Cd38*^hi^ macrophages in the fracture callus of young and aged mice, respectively.

**B**. Signal reception and transmission patterns between *Cd38^hi^* macrophages, progenitor

cells, and *Nlrp3*^hi^ neutrophils in middle-aged periosteum.

**C.** Signal reception and transmission patterns between *Cd38^hi^* macrophages, progenitor

cells, and *Nlrp3*^hi^ neutrophils in young periosteum.

**D**. Age-dependent expression of *Csf1* in progenitor cells and differential expression by progenitor subpopulations.

**E**. Representative images of macrophage migration following co-culture with either the blank control, young pSSCs, or aged pSSCs.
**F**. Quantification of migrated macrophages after co-culture with the blank control, young pSSCs, or aged pSSCs.

**G**. Differential expression of *Cxcl1* and *Cxcl12* in progenitor cells.
